# Validity of Anogenital Distance as a Marker of *in Utero* Phthalate Exposure

**DOI:** 10.1289/ehp.114-1332693

**Published:** 2006-01

**Authors:** Gerald N. McEwen, Gerald Renner

**Affiliations:** Cosmetic, Toiletry and Fragrance Association, Washington, DC, E-mail: mceweng@ctfa.org; Colipa, The European Cosmetic Toiletry and Perfumery Association, Brussels, Belgium

In their article in the August issue of *EHP*, Swan et al. (2005) purport to show that anogenital distance (AGD) in male infants is correlated with maternal phthalate exposure during pregnancy. The AGD has been shown to decrease in male newborn rats following maternal exposure to antiandrogens (Gray et al. 2001). However, little is known regarding AGD in humans, or about effects on AGD, if any, following *in utero* hormonal exposure. Furthermore, significant limitations in the study undermine the validity of the correlation reported by these authors. Some major considerations include the following.

All male infants evaluated in the study appeared normal (Swan et al. 2005). Therefore, there is no evidence for potential adverse effect in the test population. Because little is known about AGD in human infants and its variation, no conclusion can be drawn whether the reported values are normal or abnormal. The range of AGD values seen among study subjects likely represents typical biologic variation that would be expected to occur among normal study subjects.

The only available historical data on AGD in male human infants (Salazar-Martinez et al. 2004) used a different parameter for male infants (distance from anus to the base of the scrotum), which did not show a similar correlation with maternal phthalate exposure.

Swan et al. (2005) failed to take into account the phenotypes of the parents as a variable that would influence the AGD of the study subjects, much as other human anatomical parameters (i.e., height) would have a genetic component.

The study subjects varied widely in age, height, and weight. To compensate for this variability, Swan et al. (2005) defined a new parameter, which they termed the “anogenital index” (AGI), by dividing AGD by body weight. In the absence of validation, the significance of the AGI is not known, and variation cannot be assumed to be related to hormonal exposure. Swan et al. suggested that the AGI is proportional to the normal genital development of male infants, but they provided no supporting evidence. Also, much scatter can be seen in the plot of “AGI by boy’s age” (Figure 1; Swan et al. 2005). Salazar-Martinez et al. (2004) found that, in male infants,AGD correlated best with length, not weight.

Per definition, the AGD represents a one-dimensional parameter of the human anatomy. In analogy to similar anatomic parameters (e.g., length of limbs, hands, or feet), the AGD is likely to be proportional to body length and not to body weight. Therefore, Swan et al.’s use of the (body weight-related) AGI in the study has little biologic plausibility and appears to be arbitrary.

Swan et al. (2005) did not normalize maternal phthalate urinary concentrations for urine volume. This leaves open the possibility that higher urinary phthalate concentrations in individuals may have been due to lower urinary volume rather that higher phthalate exposure, and casts doubt on the maternal exposure classification categories. Phthalate levels were based on only a single sample per individual, and fetal development at the time of urine sampling was not reported.

Numerous maternal factors (alcohol consumption, medication, profession, body mass) may affect fetal development. Although it is unknown what factors, if any, would influence AGD in human infants, in the absence of these data, confounding factors cannot be excluded.

The levels of phthalates Swan et al. (2005) reported in maternal urine samples are extremely low, and the corresponding exposures are many orders of magnitude lower than the exposures at which selected phthalates have been found to have adverse reproductive effects in rodents. For example, assuming excretion of 2 L of urine/day, the reported concentration of butyl benzyl phthalate corresponds to an exposure of approximately 60 μg/day, or 1 μg/kg/day for a woman weighing 60 kg. Butyl benzyl phthalate has been shown to have only slight, hormone-like effects in rats at doses of ≥ 100 mg/kg/day (Nagao et al. 2000), or ~ 100,000-fold higher than the levels seen by Swan et al. (2005). In the case of the metabolite monoethyl phthalate, the exposure level for the corresponding parent compound diethyl phthalate was on the order of 1,000,000-fold lower than a level found to have no adverse reproductive effects in rats (4,000 mg/kg/day, the highest dose tested) (Scientific Committee on Cosmetic Products and Non-food Products 2002). It is biologically and toxicologically inconceivable that such low levels of human exposure would produce the significant structural differences claimed by Swan et al. (2005).

In summary, the relevance of AGD as an end point of interest in humans is entirely speculative, and the correlation reported by Swan et al. (2005) is lacking in biologic plausibility and remains unproven.
